# Fast and safe mapping of ventricular tachycardia in patient with left ventricular assist device

**DOI:** 10.1002/ccr3.2004

**Published:** 2019-02-19

**Authors:** Nicola Trevisi, Antonio Frontera, Federico Calore, Kenji Okubo, Paolo Della Bella

**Affiliations:** ^1^ Arrhythmology Unit Ospedale San Raffaele Milan Italy; ^2^ Abbott Medical Italy Agrate Brianza Italy

**Keywords:** ablation, left ventricular assist device, mapping, multi‐electrode, ventricular tachycardia

## Abstract

The characterization of the pathological substrate and/or mapping of the clinical ventricular tachycardia in patients with left ventricular assist device may represent a challenge, due to the risk of entrapment of the intracardiac catheter into the inflow cannula. Hereby, we present the technique of a fast and safe mapping using a 20‐poles catheter which allowed the identification of the critical isthmus during ventricular tachycardia.

## 1 CASE REPORT

**Figure 1:**
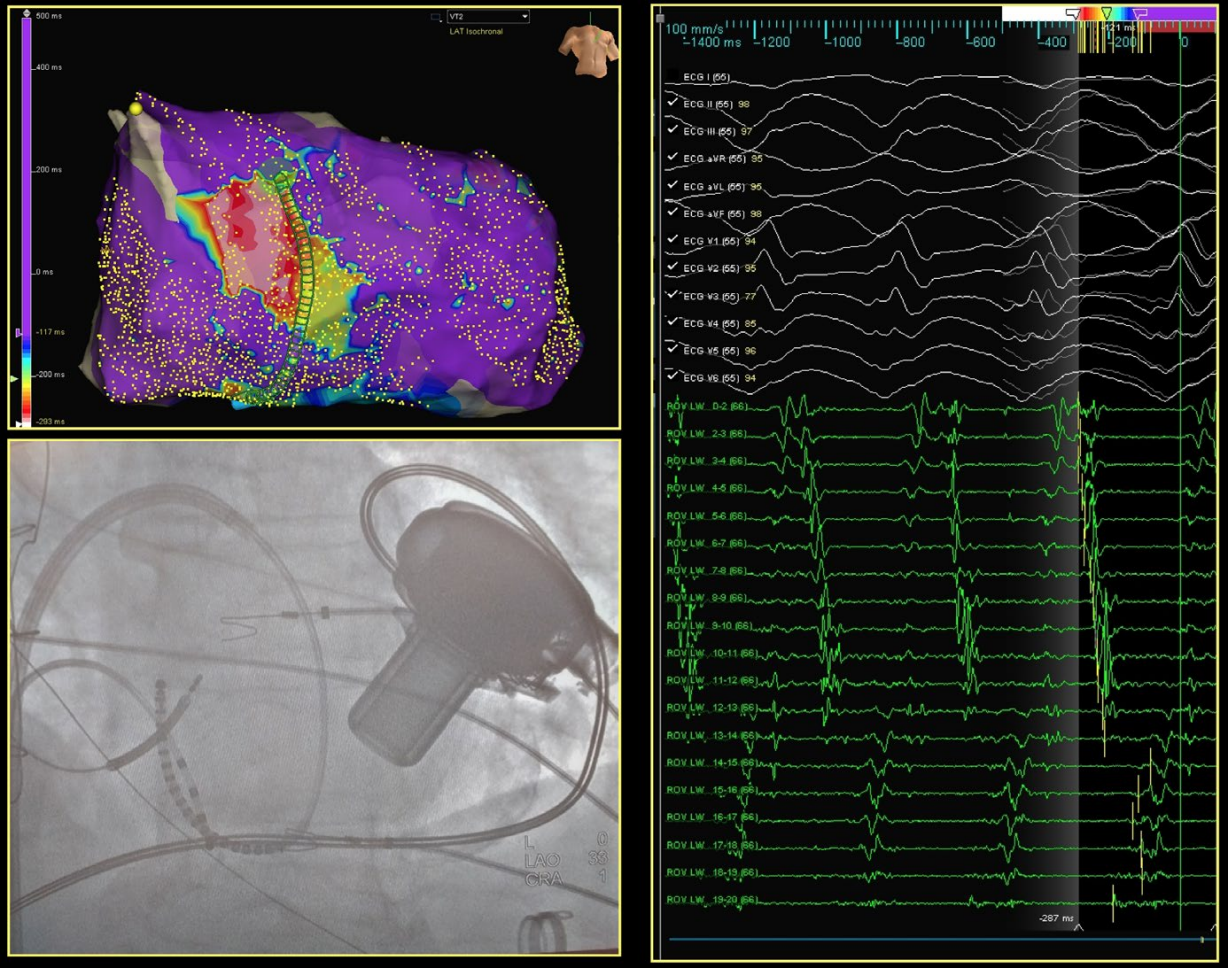
Endocardial left ventricle activation map of the clinical ventricular tachycardia obtained with Livewire™ catheter. On the upper left panel, the activation map of the ventricular tachycardia (VT), showing the diastolic propagation from white (entrance), to yellow/green (exit); on the right panel, the correspondent electrograms recorded all along the twenty poles of the catheter with the entire diastolic pathway of VT. Bottom left panel: X‐ray position of the Livewire™ catheter and its relationship with the left ventricular assist device

We describe the case of a 70‐year‐old man who presented at our institution for recurrent episodes of ventricular tachycardia (VT) refractory to amiodarone and mexiletine in the weeks following a left ventricular assist device (HeartWare Inc, Framingham, MA, USA) procedure. The patient had a history of anterior myocardial infarction with severe left ventricular dysfunction (EF 20%). Under general anesthesia, left ventricle was approached via trans‐septal puncture. Using a 20‐poles catheter (2‐2‐2 mm interelectrodes distance, Livewire™, Abbott, MN, USA), a high‐density map was built with the EnSite Precision™ Mapping System (Abbott, MN, USA). During sinus rhythm, a large myocardial scar (<0.2 mV) on the anterior and septal LV wall was documented, but no late potentials were recorded. A clinical VT (CL [cycle length] 400 ms), hemodynamically tolerated, was induced. An activation map of the VT was attempted (Figure 1) with the entire diastolic pathway depicted. Pulses of RF (50 W, 43°C) were delivered at the isthmus site with immediate termination of the arrhythmia. No ventricular arrhythmias were induced up to three extrastimuli. No complications were encountered. At 6 months of follow‐up, patient did not experience VT recurrences.

## 2 DISCUSSION

Ventricular tachycardia recurrences often complicate the course of patients with left ventricular assist device (LVAD). Transcatheter ablation, as shown by a recent multi‐center study,^1^ represents an effective and safe procedure. However, ventricular mapping in such patients with LVAD may represent a challenge, due to the risk of entrapment of the intracardiac catheter into the inflow cannula. Owing to this potential issue, multi‐electrodes mapping tools (and their multi‐splines) are not usually used in such patients, although they might provide substantial advantages especially during mapping of incessant VT and large myocardial scar. Here, we present the case of a VT circuit depicted with the limitation of this catheter (no splines, linear shape, no conformability to the ventricular wall) allowing a safe, extensive, and rapid mapping. Furthermore, the automatic acquisition of very low amplitude signals has not been influenced by the presence of LVAD. This approach represented an efficient, time, and cost saving alternative in such complex cases.

## CONFLICT OF INTEREST

Dr Paolo della Bella is consultant for Abbott and Biosense webster companies. Research grant from Biotronik, Biosense Webster, Abbott and Boston Scientific.

## AUTHOR CONTRIBUTION

NT and KO: performed the case and helped to draft the manuscript. AF, FC, PDB: acquired, analyzed the data, and drafted the manuscript.
